# NSs Encoded by Groundnut Bud Necrosis Virus Is a Bifunctional Enzyme

**DOI:** 10.1371/journal.pone.0009757

**Published:** 2010-03-18

**Authors:** Bhushan Lokesh, Panigrahi R. Rashmi, Bhat S. Amruta, Dharmaiah Srisathiyanarayanan, Mathur R. N. Murthy, Handanahal S. Savithri

**Affiliations:** 1 Department of Biochemistry, Indian Institute of Science, Bangalore, Karnataka, India; 2 Molecular Biophysics Unit, Indian Institute of Science, Bangalore, Karnataka, India; Institut Pasteur Korea, Republic of Korea

## Abstract

*Groundnut bud necrosis virus* (GBNV), a member of genus *Tospovirus* in the family *Bunyaviridae*, infects a large number of *leguminosae* and *solanaceae* plants in India. With a view to elucidate the function of nonstructural protein, NSs encoded by the small RNA genome (S RNA), the NSs protein of GBNV- tomato (Karnataka) [1] was over-expressed in *E. coli* and purified by Ni-NTA chromatography. The purified rNSs protein exhibited an RNA stimulated NTPase activity. Further, this activity was metal ion dependent and was inhibited by adenosine 5′ (β, γ imido) triphosphate, an ATP analog. The rNSs could also hydrolyze dATP. Interestingly, in addition to the NTPase and dATPase activities, the rNSs exhibited ATP independent 5′ RNA/DNA phosphatase activity that was completely inhibited by AMP. The 5′ α phosphate could be removed from ssDNA, ssRNA, dsDNA and dsRNA thus confirming that rNSs has a novel 5′ α phosphatase activity. K189A mutation in the Walker motif A (GxxxxGKT) resulted in complete loss of ATPase activity, but the 5′ phosphatase activity was unaffected. On the other hand, D159A mutation in the Walker motif B (DExx) resulted in partial loss of both the activities. These results demonstrate for the first time that NSs is a bifunctional enzyme, which could participate in viral movement, replication or in suppression of the host defense mechanism.

## Introduction

The genus *Tospovirus* of the family *Bunyaviridae* is unique in infecting plants whereas the other members of this family infect only animals. Tospoviruses are transmitted by thrips in a persistent manner. They are quasi spherical enveloped viruses of 80–120 nm diameter with tripartite single stranded RNA genomes. The largest of the three single stranded RNA molecules, L RNA, codes for RNA dependent RNA polymerase in the virion complementary sense. The middle (M) RNA encodes the precursor for glycoproteins G1 and G2 in the virion complementary sense and the non structural protein, NSm in the virion sense orientation. The small (S) RNA codes for the nonstructural protein, NSs in the virion sense and nucleocapsid protein N in the virion complementary sense [2]. Using a green fluorescent protein based transient suppression assay, it was shown that NSs from *Tomato spotted wilt virus* (TSWV) (type member of *Tospovirus*) could function as a suppressor of post transcriptional gene silencing (PTGS) [3]. NSs is also associated with the symptom severity [4] and is known to form the paracrystalline array in the cytoplasm of infected plant and thrips [5]. However, biochemical characterization of NSs has not been reported so far. Among Tospoviruses reported from India, *Groundnut bud necrosis virus* (GBNV) also called *Peanut bud necrosis virus* (PBNV) has been well characterized [6], [7], [8], [9], [10]. The N and NSm genes of several isolates from different locations in India infecting *Leguminosae* and *Solanaceae* plants have been sequenced and shown to be strains of GBNV [8], [9], [11], [12], [13], [14]. We have earlier purified and characterized a strain of GBNV infecting tomato in Karnataka GBNV- To (K) [1]. In the present study, we have cloned the NSs gene of GBNV-To (K) and overexpressed it in *E.coli*. The amino acid sequence of NSs protein contains Walker A (GxxxxGKT) and Walker B (DExx) motifs. The proteins which posses the Walker A and Walker B motifs exhibit RNA/DNA stimulated NTPase activity[15]. Some of the viral encoded non structural proteins such as NS3 protein from *West Nile flavivirus*, *Hepatitis C virus*, *Yellow fever virus* and *Dengue virus type 2* [16], [17], [18], which have these motifs are shown to exhibit nucleic acid stimulated NTPase activity. However, none of the viral encoded proteins of the *Bunyaviridae* family have been shown to exhibit the NTPase activity. Therefore, it was of interest to examine, if the NSs possessed the NTPase activity. The rNSs exhibited poly(A)-stimulated metal ion dependent NTPase/dATPase activity. Further it was shown to exhibit ATP independent 5′ DNA/RNA phosphatase activity. Mutation of K189 to A in the Walker motif A resulted in the complete loss of ATPase activity but not the 5′ phophatase activity, whereas mutation of D159 to A in the Walker motif B resulted in the partial loss of both the activities. Thus, for the first time we demonstrate that NSs is a bifunctional enzyme and it might play a pivotal role at different stages of viral life cycle.

## Materials and Methods

The fine chemicals used for biochemical and molecular biological work were purchased from Sigma, Calbiochem and Novagen. Restriction endonucleases and DNA modification enzymes and polymerases were purchased from New England Biolabs and MBI Fermentas. Radioactive isotopes were purchased from PerkinElmer Life Sciences. All the other chemicals used were of analytical grade.

### Cloning and over-expression of NSs gene of GBNV-To (K)

The NSs gene of GBNV-To (K) was amplified by RT-PCR using partially purified viral RNA as template and specific sense and anti-sense primers, designed on the basis of the genomic sequence of S RNA of GBNV (accession number U27809, table 1). The PCR product was digested with the restriction enzymes *Nhe*I and *Xho*I (purchased from MBI Fermantas) and ligated into the corresponding sites of plasmid pRSET-C (purchased from Invitrogen). The plasmid was named pRSETC-NSs and propagated in *E. coli*. The NSs coding sequence was confirmed by DNA sequencing. The recombinant NSs clone was transformed into C43 (DE3) *E.coli* cells and over-expressed by induction with 0.3 mM IPTG (purchased from Sigma) for 3 hours at 37°C. The cell pellet was resuspended in buffer A (20 mM Tris-HCl pH 8.0 and 300 mM NaCl), sonicated and centrifuged at 12000 rpm for 15 min. The supernatant and pellet fractions were analyzed by 12% SDS-PAGE. The rNSs protein was purified from the soluble fraction.

**Table 1:** List of the primers used for full length NSs gene amplification and mutagenesis, s - sense primer, a - antisense primer.

Name	5′ to 3′ sequence	Description
**NSs-s**	CTAGCTAGCCATATGTCAACTGCAAAGAATGC	Sense primer used to amplify the full length of NSs gene. Under lined sequence corresponds to *Nhe*I and *Nde*I restriction sites.
**NSs-a**	CCCTCGAGGGTTATTCTGCTTTCACAATGAAGTG	Primer corresponding to 3′ end of NSs gene in the antisense orientation. Under lined sequence corresponds to *Xho*I restriction site.
**NSs K189A-s**	CTGTTATGGGAGCGACAACATCCTACTGGAGAG	Sense primer used for mutation of K189 in Walker A motif to A.
**NSs K189A-a**	CTCTCCAGTAGGATGTTGTCGCTCCCATAACAG	Antisense primer used for mutation of K189 to A
**NSs D159A-s**	CCCTCCGGATGGTATCAAGCTGAATGCTG	Sense primer used for mutation of D159 in Walker B motif to A. Under lined sequence corresponds to *Kpn*21 restriction site.
**NSs D159A-a**	CAGCATTCAGCTTGATACCATCCGGAGGG	Antisense primer used for mutation of D159 to A. Under lined sequence corresponds to *Kpn*21 restriction site.

All the mutations were confirmed by DNA sequencing.

### Purification of rNSs by Ni-NTA affinity chromatography

The rNSs with its N-terminal histidine tag was purified by the Ni–NTA chromatography. C43 *E.coli* cells harboring the pRSETC-NSs clone were cultured in LB medium (2L) containing ampicillin (50 µg/ml) at 37°C. The expression of rNSs was induced, when the O.D. at 600 nm reached 0.6, by the addition of IPTG (0.3 mM) and the cells were cultured further for 4 hours at 37°C. The cells were harvested, re-suspended in buffer A and sonicated till the suspension became optically clear. The cell lysate was then spun down at 10000 rpm for 10 minutes. To the supernatant, Ni-NTA resin (500 µl/500 ml of culture) was added and left for 2 hours in an end-to-end rotor for binding. The beads were allowed to settle and the unbound supernatant discarded. The beads were washed once with buffer A and then twice with buffer A containing 20 mM imidazole. The rNSs was eluted with 5 ml buffer A containing 250 mM imidazole pH 8.0. The eluted fractions were analyzed by SDS-PAGE [19]. The fractions containing rNSs were pooled and the protein was dialyzed against 20 mM Tris-HCl pH 8.0 buffer containing 100 mM NaCl. The protein obtained by this method was reasonably pure and was suitable for further characterization. The molecular mass of the purified rNSs was determined by matrix-assisted laser desorption/ionization (MALDI) mass spectrometer.

### Site-directed mutagenesis

The site directed mutagenesis was carried out by PCR based method [20] using appropriate sense and anti sense primers with desired changes. The oligonucleotide primers used for site-directed mutagenesis (SDM) were custom made from Sigma. Mutagenic DNA primers (table 1) were designed to create alanine substitution at residues K189 and D159 in GxxxxGKT and DExx motifs of rNSs respectively. The presence of the desired mutations was screened initially by the gain or loss of a restriction site and was confirmed by DNA sequencing.

### Circular dichroism spectroscopy

The far UV CD spectrum of rNSs protein was recorded using a Jasco-815 spectropolarimeter. The ellipticity was monitored at 25°C from 200 nm to 250 nm using 0.2 mg/ml of the purified protein in 20 mM Tris-HCl pH 8.0 and 100 mM NaCl. A scan speed of 50 nm/min, 0.2 cm path length cuvette, band width of 1 nm and response time of 1 s were used. The average of three spectra was taken and corrected with buffer blanks. The molar ellipticity was calculated using the software provided by the manufacturer.

The thermal stability of rNSs was monitored in a Jasco-815 spectropolarimeter fitted with Peltier thermal control system (PTC-423S). 0.2 mg/ml of the purified protein in 20 mM Tris-HCl pH 8.0 and 100 mM NaCl was heated from 10°C to 100°C. The change in molar ellipticity at 222 nm was monitored as a function of temperature to obtain the thermal melting curve. The ellipticity was measured at 222 nm using 0.2 mg/ml protein in a 0.1 cm path length cuvette with band width of 1 nm and response time of 1 sec. The temperature was raised at a rate of 2°C/minute from 10–100°C.

### Fluorescence spectroscopy

The fluorescence spectra were recorded in a PerkinElmer LS5S luminescence spectrometer. The intrinsic fluorescence spectrum was monitored from 300–400 nm upon excitation at 280 nm in a 0.5 cm path length cuvette. The concentration of the protein used was 0.1 mg/ml.

### NTPase assay

rNSs (0.4, 0.8, 1.6 and 2 µg) was incubated at 25°C with 0.1 µCi of labelled nucleotides [γ-^32^P] ATP or [γ-^32^P] GTP (purchased from PerkinElmer Life Sciences) or [α-^32^P] UTP, CTP, or dATP (purchased from PerkinElmer Life Sciences) in 50 mM MOPS buffer pH 7.0 containing 2 mM MgCl_2_, 2 mM dithiothreitol, 0.5 mg of bovine serum albumin per ml, in the presence or absence of 0.2 mg per ml poly(A) for 30 min. and the reaction was stopped by the addition of 100 mM EDTA (1 µl). The total volume of the reaction mixture was 10 µl. For kinetic studies, initially a stock of cold NTPs containing 0.008 µCi of ^32^P labelled respective NTP was prepared and the substrate concentration was varied from 100–1000 µM for ATP and 10–80 µM for CTP, GTP, UTP and 2.5–20 µM for dATP. The reaction mixture was incubated with 2 µg of rNSs protein in the assay buffer. To determine the kinetic parameters of D159A mutant NSs, 10–70 µM ATP was used with 2 µg of mutant protein. Three independent sets of experiments were carried out to determine the kinetic parameters. Substrate hydrolysis was measured by Imagegauge software (Fujifilm) and the enzyme activity (v) was calculated using the formula given below:
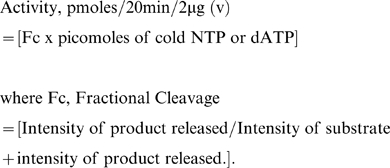



The Pi released or α-NDP/dADP released was measured when [γ-^32^P] ATP/GTP or [α-^32^P] NTP/dATP was used as tracer respectively.

The *K_M_* and *k*_cat_ values were calculated from the Lineweaver-Burk plot (1/v vs 1/s).

### Thin layer chromatography

Reaction products of NTPase/dATPase and phosphatase assays were analyzed by thin layer chromatography using polyethyleneimine-cellulose (PEI cellulose F) plates (purchased from Merck). For this, the chromatographic chamber was saturated with developing solution of formic acid (1 M), EDTA (1 mM) and LiCl_2_ (0.5 M) for two hours. 0.5 µl reaction mixture was spotted on the PEI cellulose plate and the plate was kept in saturated chromatographic chamber for developing the chromatogram. The intensity of the spots was measured using a phosphor imager.

### Phosphatase assay

The substrates for the phosphatase reaction were 5′ end labeled using [γ-^32^P] ATP and T4 polynucleotide kinase according to manufacturer's protocol. Free nucleotides were removed from the end labelled substrate using Sephadex G25 spun column.

rNSs (1.5 µg) was incubated at 25°C for 30 min with 5′ labeled ssDNA (K189A sense primer, 0.5 nM), ssRNA {poly(A), 4 nM}, dsDNA (1 nM) and dsRNA (0.2–1 nM) in 50 mM MOPS buffer pH 7.0 containing 2 mM magnesium chloride, 2 mM dithiothreitol, 0.5 mg of bovine serum albumin per ml. The reaction was stopped by addition of 100 mM EDTA (1 µl) in a total volume of 10 µl and release of Pi was measured as described above.

### Preparation of dsDNA and dsRNA substrates for 5′ phosphatase activity

dsDNA was made by annealing of sense and antisense primers (D159A). Resulting annealed product was end labelled using [γ-^32^P] ATP as described above. Further, dsDNA was gel eluted from the mixture of dsDNA and ssDNA. dsRNA was generated by annealing of *in vitro* transcribed sense and antisense 1 kb *Sesbania mosaic virus* RNA transcripts (kind gift from K. Govind). dsRNA was separated from ssRNA on 1% Agarose gel and dsRNA was gel eluted. The concentration of dsDNA/dsRNA was determined by measuring the O.D. at 260 nm in a spectrophotometer.

### Immunodepeletion

Purified rNSs (2 µg) was incubated with polyclonal antibodies raised in rabbit against rNSs at 4°C for two hours. The rNSs-antibody complex was precipitated by the addition of protein A sepharose beads. The ternary complex was separated by centrifuging the mixture at 5,000 rpm for two minutes and the supernatant was tested for ATPase activity.

## Results

### Bioinformatic analysis of GBNV-To (K) NSs protein

**Figure 1:**
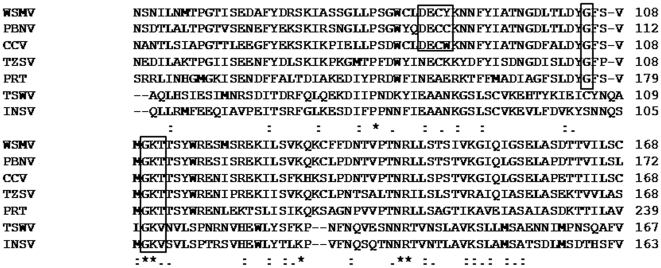
The multiple sequence alignment of the NSs protein of six tospoviruses. Multiple sequence alignment of NSs from different species of tospoviruses was performed using Clustal W program (http://www.ebi.ac.uk/Tools/clustalw/). Sequence data were from the Genbank database, and accession numbers are as follows: *Watermelon silver mottle virus*, NP_620770; *Peanut bud necrosis virus* (PBNV), ABC59432; *Capsicum chlorosis virus* (CCV), ABO72544; *Tomato zonate spot virus* (TZSV), YP_001740043; *Polygonum ringspot tospovirus* (PRT), ABO31116.1; *Tomato spotted wilt virus* (TSWV), ABI94070; *Impatiens necrotic spot virus* (INSV), ABW79860. The amino acid sequences of the Walker A and B motifs are boxed.

In order to establish the role of NSs during the viral life cycle, bioinformatic analysis was performed to identify the motifs present. Supplementary Fig. S1. shows the putative amino acid sequence of GBNV-To (K) NSs along with secondary structure prediction. The protein was predicted to have 27% α helix, 25% β strand and 48% random coil. Motif search using the expasy server showed the presence of Walker motif A and B (Fig. S1). It may be noted that, in most ATPases Walker A motif precedes Walker B motif. However, in the NSs protein, the two motifs are reversed. Multiple sequence alignment of GBNV-(To) K NSs protein with six different tospoviruses representing different species revealed that glycine (G188) and lysine (K189) residues of Walker motif A are very well conserved across the T*ospovirus* genus (Fig. 1). In TSWV and *Impatiens necrotic spot virus* (INSV), which show the lowest percent identity with PBNV (19% and 17%, respectively) also these residues are conserved, even though the canonical Walker A (GxxxxGKT) motif is not present in these two viruses. On the other hand, walker motif B (DExx) is conserved only in PBNV, *Watermelon silver mottle virus* (WSMV) and *Capsicum chlorosis virus* (CCV) (Fig. 1).

### Purification and charaterization of rNSs

**Figure 2:**
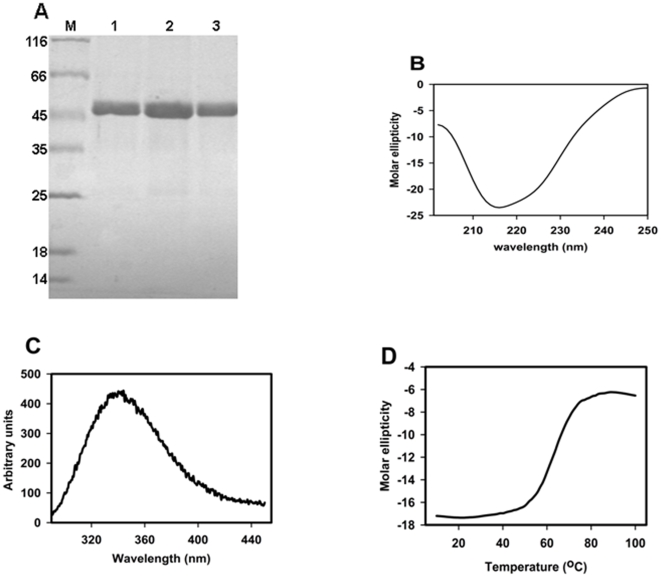
Purification and biophysical characterization of rNSs. (**A**) Purification of rNSs. rNSs was purified from the IPTG induced cells transformed with pRSETC-NSs by Ni-NTA chromatography. The purified protein was analyzed by 12% SDS PAGE. The gels were stained with Coomassie blue. Lane M molecular mass marker proteins (in kDa); lane 1–3 individual fractions eluted from Ni-NTA column. (**B**) CD spectrum of rNSs. The CD spectrum of rNSs (0.2 mg/ml) was recorded using Jasco-815 spectropolarimeter. The molar ellipticity was calculated using subunit mass of 52 kDa. (**C**) Fluorescence spectrum of rNSs. The fluorescence spectrum of rNSs (0.1 mg/ml) was recorded using Perkin Elmer LS5S luminescence spectrometer after excitation at 280 nm. (**D**) Thermal melting of rNSs. The molar ellipticity (Y-axis) of rNSs was measured at 222 nm, as a function of temperature (X-axis) and plotted as shown.

The GBNV-(To) K NSs gene was cloned into pRSET-C vector and overexpressed, as described in the materials and methods section, in C43 (DE3) *E.coli* cells. rNSs was purified from the soluble fraction by Ni-NTA affinity chromatography and the purity of the protein was checked by SDS-PAGE analysis. A single band corresponding to the expected molecular mass of 52 kDa was observed in the eluted fractions (Fig. 2A lane 1–3). The yield of the purified protein was 7–10 mg from 500 ml culture. The molecular mass of rNSs was confirmed by mass spectrometric analysis. The molecular mass thus obtained was 51810 Da, which agreed with that predicted from the sequence of histidine-tagged rNSs (52 kDa).

The Circular dichroism spectra analysis of rNSs showed a minimum in the range of 215–223 nm (Fig. 2B). The intrinsic fluorescence emission spectrum showed a maximum at 338 nm upon excitation at 280 nm (Fig. 2C). rNSs contains five tryptophans and eleven tyrosines which are buried as is apparent from the emission fluorescence maximum at 338 nm. These results demonstrate that rNSs is in a folded conformation. The thermal stability of rNSs monitored by CD spectroscopy revealed that it is a stable protein with a Tm of 65°C (Fig. 2D).

### ssRNA-stimulated ATPase activity

**Figure 3:**
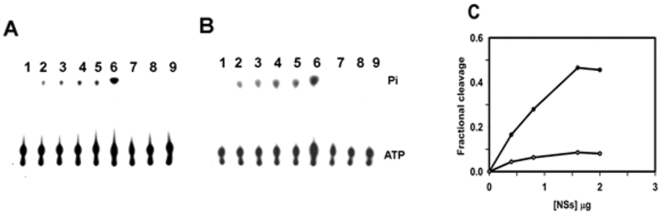
ATPase activity of rNSs with and without poly(A). (**A**) ATPase assay in the absence of poly(A) and (**B**) ATPase assay in the presence of poly(A) (0.2 mg/ml). rNSs was incubated with 0.1 µCi of [γ-^32^P] ATP at 25°C for 30 min. in 50 mM MOPS buffer pH 7.0 containing 2 mM MgCl_2_, 2 mM DTT, 0.5 mg bovine serum albumin per ml in the presence or absence of poly(A) (0.2 mg/ml). Total volume of the reaction mixture was 10 µl. The reaction product was analysed by spotting 0.5 µl of the reaction mixture on the PEI cellulose plate and the chromatogram was developed in formic acid (1 M), EDTA (2 mM), and LiCl_2_ (0.5 M). Lane 1: γ-^32^P ATP alone. Lane 2–5: γ ^32^P ATP with increasing concentration of rNSs (0.4, 0.8, 1.6 and 2 µg respectively). Lane 6: positive control RecoP15I, an ATP dependent restriction endonuclease was used instead of rNSs, Lane 7: immunodepleted rNSs, Lane 8: reaction carried out with hexahistidine tagged-AV2 (2 µg) instead of rNSs. Lane 9: Reaction carried out in presence of 10 mM EDTA. (**C**) Quantitation of ATPase activity of rNSs. The plate was autoradiographed after drying. The substrate hydrolysis was measured using the Image gauge software. The fractional cleavage was calculated as [Intensity of released product/Intensity of uncleaved substrate + Intensity of released product]. The fractional cleavage of ATP was calculated and plotted as a function of the amount of protein. Each point represents the average of two experiments.

ATPase activity was assayed as described in the methods section using [γ-^32^P] ATP as the substrate. The radiolabelled Pi released was monitored by TLC on PEI cellulose plates. As mentioned earlier, proteins which posses the Walker A and Walker B motifs are known to exhibit the RNA/DNA stimulated NTPase activity [15]. Therefore, purified rNSs (0.4–2 µg) was used to determine the ATPase activity in the absence and presence of Poly (A). As shown in Fig. 3, rNSs could hydrolyze [γ-^32^P] ATP in a concentration-dependent manner. The activity was higher in the presence of poly(A) (Fig. 3B). The product of ATP hydrolysis by rNSs had the same mobility as the phosphate released by RecoP51 ATPase which was used as the positive control (Fig. 3A and B, lane 6). In contrast, another viral protein from the *Cotton leaf curl virus*, His tagged-AV2, did not show the release of phosphate (Fig. 3A and B, lane 8), suggesting that the activity was not due to the histidine tag present at the N-terminus of rNSs. Further, no release of phosphate was detected following immunodepletion of rNSs, confirming that the activity was inherent to the NSs protein (Fig. 3A and B, lane 7). The reaction was completely inhibited by 10 mM EDTA (Fig. 3A and B, lane 9) suggesting that the reaction is metal-ion dependent. The amount of product (phosphate) released was quantitated as described in the methods section. There was a 10-fold increase in the activity in the presence of poly(A) as compared to that without poly(A) (Fig. 3C). The reaction was linear in the presence of poly(A) upto 1.6 µg of rNSs (Fig. 3C). Since the ATPase activity of rNSs was much higher in the presence of poly(A), all further standardization of reaction conditions was carried out in the presence of poly(A). The product release was linear up to 30 mins (Fig. S2A) and the ATPase activity of rNSs was enhanced by the addition of MgCl_2_. Maximum activity was observed at 2.5 mM of MgCl_2_ (Fig. S2B). Addition of increasing concentrations of EDTA lead to inhibition of activity and the ATP cleavage was completely inhibited at 5 mM EDTA (Fig. S2C). The pH profile was a typical bell shaped curve with a distinct pH optimum at pH 7.0 (Fig. S2D) and the optimum temperature of the reaction was 25°C (data not shown).

### Substrate specificity

**Figure 4:**
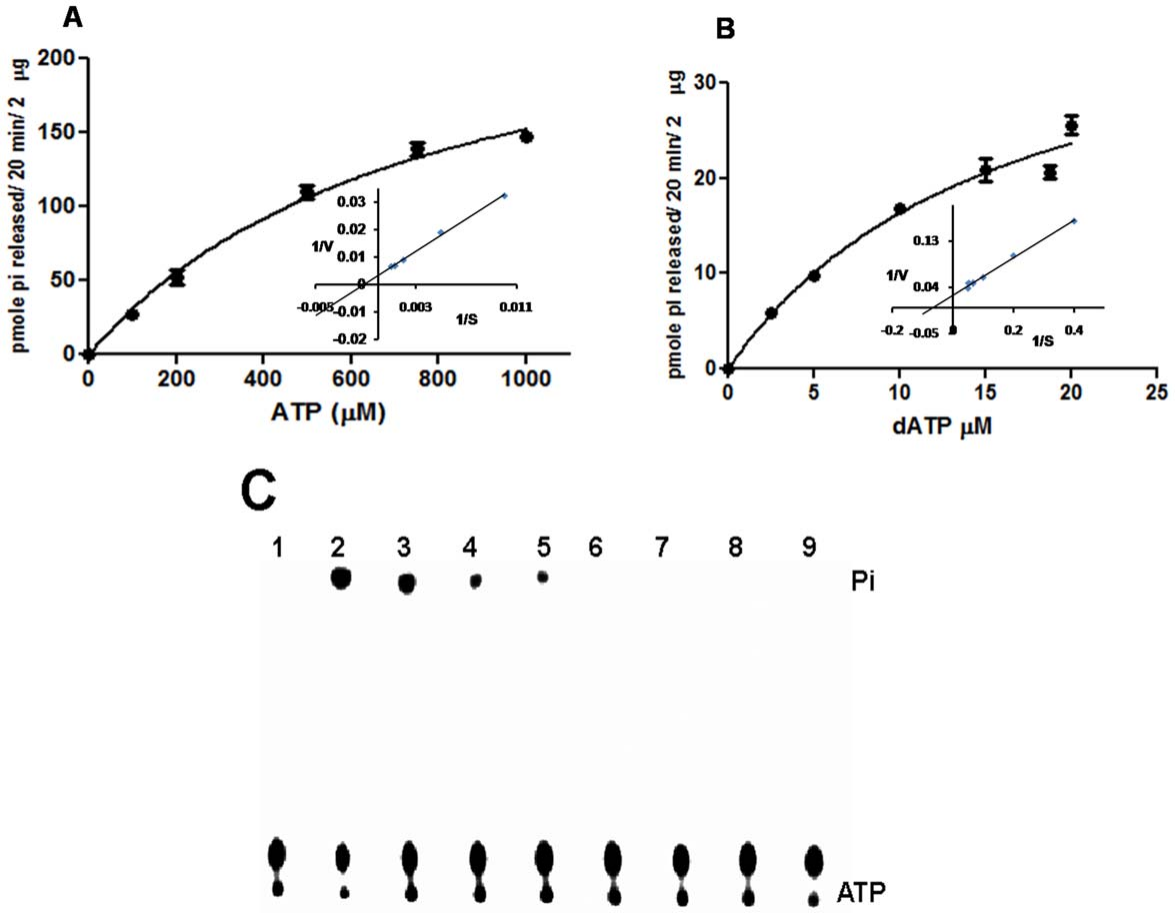
Substrate saturation curve and Lineweaver-Burk plot for the wild type rNSs with ATP and dATP. (**A**) Substrate saturation curve and Lineweaver-Burk plot for the wild type rNSs with ATP and (**B**) with dATP as substrates. A stock of cold NTPs containing 0.008 µCi of ^32^P labeled respective NTP was prepared and the substrate concentration was varied from 100–1000 µM for ATP and 10–80 µM for dATP as shown in Fig. 4 (A and B respectively). The reaction mixture was incubated with 2 µg of NSs protein in the assay buffer for 20 min at 25°C. The Pi/dADP released was analyzed by TLC using PEI cellulose F plate and enzyme activity (v) was calculated using the formula given below: Activity, pmoles/20 min/2 µg (v)  =  [Fc x picomoles of cold NTP/dATP] where Fc, Fractional Cleavage  =  [Intensity of product released/Intensity of substrate + intensity of product released.]. The Lineweaver-Burk plot (inset Fig. 4A and B) was generated by plotting 1/velocity vs 1/substrate. (**C**) ATPase activity of rNSs in the presence of ATP analog adenosine (β, γ imido) triphosphate. ATPase activity was assayed at various concentrations of ATP analog as described in the materials and methods section with 1.6 µg of rNSs and 5 nM of [γ-^32^P] labelled ATP. Lane 1: negative control (-rNSs), Lane 2–3: no ATP analog added, Lane: 4–9, Increasing concentration of ATP analog (1, 5, 10, 15, 20, 25 mM respectively).

The specificity of cleavage of nucleotide triphosphate by rNSs was examined using [γ-^32^P] labelled ATP and GTP or [α-^32^P] CTP, UTP and dATP as substrates. The activity was monitored by TLC as described earlier. The amount of product (NDP or Pi) released was quantitated and the substrate saturation curves as well as Lineweaver-Burk plots were drawn for each of the substrates as described in material and methods section. Fig. 4A depicts the substrate saturation curve with ATP as the substrate. Inset to Fig. 4A shows the Lineweaver-Burk plot of the same data. The *K_M_* and *k*_cat_ values were calculated to be 515.2 µM and 10.8×10^−3^ sec^−1^ respectively. Similar experiments were performed with the other NTPs. rNSs was able to hydrolyze all the NTPs and the *K_M_* and *k*_cat_ values obtained are presented in table 2. Although the *k*_cat_ value was the highest with ATP, the *K_M_* was also increased by an order of magnitude compared to other NTPs.

**Table 2:** Kinetic parameters were determined by three independent experiments.

Kinetic parameter	ATP	CTP	GTP	UTP	dATP	D159A
***K_M_*** (µM)	515.2±69	44.4±8.4	46.3±7.8	34.2±12.1	15.5±3.5	31.7±2.5

It was of interest to examine whether rNSs could cleave dATP. As shown in Fig. 4B, rNSs could also use dATP as substrate and the kinetic parameters obtained from the Lineweaver-Burk plot (Fig. 4B inset) are also presented in table 2. The *k*_cat_ for dATP was approximately 10 times lower than that of ATP, although the *K_M_* (15.5 µM) was much lower. The specificity of rNSs was also tested by using the ATP analog, adenosine 5′ (β, γ imido) triphosphate. 5 mM concentration of this ATP analog resulted in considerable decrease in the activity (Fig. 4C lane 4 and 5). Further increase in the concentration of the analog abolished the product formation completely (Fig. 4C lane 6 to lane 9).

### Mutational analysis of rNSs

**Figure 5:**
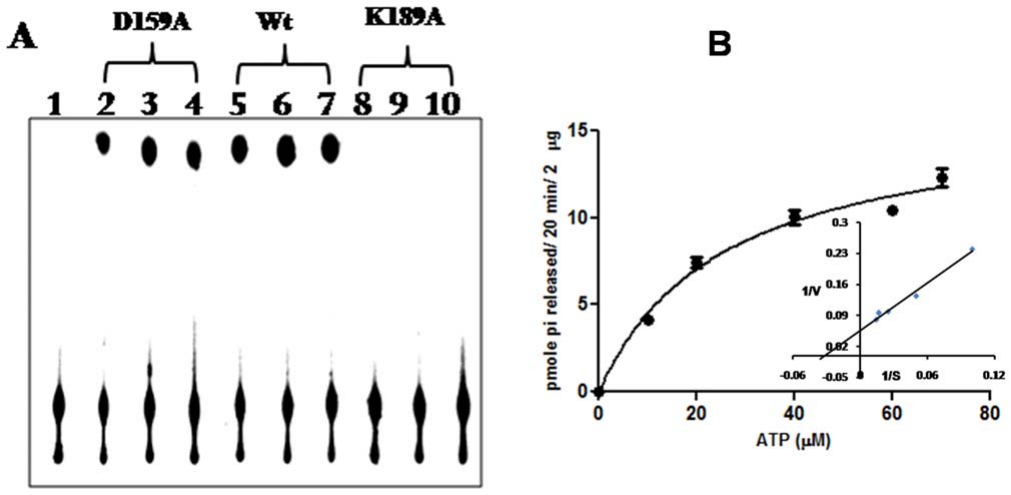
ATP hydrolysis by wild type, D159A and K189A rNSs mutants as a function of protein concentration. (**A**) The ATPase activity was tested using 0.4, 0.8 and 1.2 µg of D159A mutant (lane 2, 3 and 4 respectively) or wt rNSs (lane 5, 6 and 7 respectively) or K189A mutant lane 8, 9 and 10 respectively), Lane 1: negative control, without rNSs. (**B**) Substrate saturation curve for D159A mutant rNSs. The experiment was performed as described in the legend to fig. 4. The inset represents the Lineweaver-Burk plot of the data.

The results presented thus far clearly demonstrate that rNSs exhibits poly(A) stimulated NTPase/dATPase activities. As mentioned earlier, rNSs has sequence motifs (Walker A and Walker B) that are typical of NTP binding proteins (Fig. 1). It was of interest to examine the role of these motifs in the NTP/dATPase function of rNSs. It was shown earlier that the lysine residue of Walker motif A is crucial for NTP binding whereas aspartate and glutamate of Walker motif B coordinate the Mg^2+^ ion [21]. Therefore, K189 and D159 of the Walker A (GxxxxGKT) and B (DExx) motifs respectively in rNSs were mutated to alanine by site directed mutagenesis as described in the methods section. The K189A and D159A rNSs mutants were over-expressed and purified by Ni-NTA chromatography as described for the wild type rNSs. The purified mutant proteins were analysed on a 12% SDS-PAGE and the proteins were nearly homogeneous. The ATPase activities of the mutants were tested using 0.4, 0.8 and 1.2 µg of the protein along with the wild type rNSs as the control. As shown in Fig. 5A, the K189A rNSs mutant was completely inactive whereas the D159A mutant was partially active. Kinetic analysis of D159A mutant NSs (Fig. 5B) revealed that the *k*_cat_ was reduced by 28 fold (table 2) compared to wild type rNSs.

### 5′ phosphatase activity of rNSs

**Figure 6:**
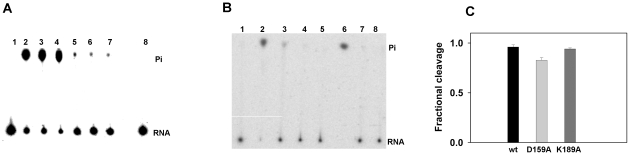
5′ Phosphatase activity of rNSs. (**A**) 5′ Phosphatase activity of rNSs (1 µg) was assayed with ssRNA {poly(A)}, as described in the methods section in the absence (lane 2–4, triplicate) and presence of 1 mM ATP (lane 5–7), Lane 8: ssRNA treated with 5 units of ATPase enzyme. (**B**) 5′ Phosphatase activity of rNSs with and without AMP. Lane 1: labelled ssRNA alone, Lane 2: ssRNA incubated with rNSs without AMP, Lane 3–5: ssRNA incubated with rNSs with increasing concentration of AMP (1, 2 and 3 mM), Lane 6: Calf intestinal alkaline phosphatase (CIP) treated ssRNA, Lane 7: ssRNA incubated with immunodepleted rNSs, Lane 8: ssRNA incubated with heat denatured rNSs (**C**) phosphatase activity of wild type, D159A and K189A mutant rNSs proteins. The activity was determined as described in methods section and fractional cleavage was estimated as described in the legend to Fig. 3. The fractional cleavage by the three proteins (1 µg) are shown as bar diagram.

**Figure 7:**
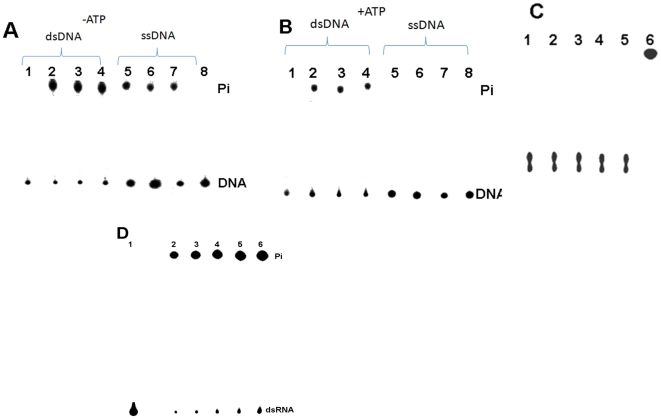
5′ Phosphatase activity of rNSs with DNA substrates. **Panel A:** The 5′ phosphatase activity of rNSs was assayed in the absence of ATP as described in the methods section. Lane 1: 5′ end labeled dsDNA (0.3 nM) alone, lane 2–4: dsDNA treated with 1 µg of rNSs (in triplicate), Lane 5–7: 5′ end labeled ssDNA treated with 1 µg of rNSs (in triplicate), lane 8: ssDNA alone. **Panel B:** The 5′ phosphatase activity of rNSs was assayed in the presence of ATP (1 mM) as described in the methods section. Lane 1: 5′ end labeled dsDNA treated with 5 units (u) of ATPase enzyme, Lane 2–4: 5′ end labeled dsDNA treated with 1 µg of rNSs (in triplicate), Lane 5–7: 5′ end labeled ssDNA treated with 1 µg of rNSs (in triplicate), Lane 8: 5′ end labeled ssDNA treated with 5 u of ATPase enzyme. **Panel C** The 5′ phosphatase activity of rNSs was assayed with 5′ end labeled siRNA substrate. Lane 1: siRNA alone, Lane 2–5: siRNA treated with increasing concentrations rNSs (1, 2 and 3 µg), Lane 6: CIP treated siRNA. **Panel D** Effect of dsRNA concentration: rNSs (0.5 µg) was incubated with increasing concentrations of 5′ labeled dsRNA and the activity was measured as described in the methods section. Lane 1: labelled dsRNA alone, Lane 2–6 rNSs incubated with 0.2, 0.4, 0.6, 0.8 and 1 nM dsRNA respectively.

The ATPase activity of rNSs is stimulated by poly(A). It was of interest to examine the activity with 5′ phosphorylated poly(A). For this, poly(A) was 5′ end labelled using T4 polynucleotide kinase (MBI fermentas) and [γ-^32^P] ATP according to the manufacturer's protocol. The 5′ end labelled poly(A) was purified using Sephadex G25 column and run on 6% native PAGE to confirm that the contaminating free [γ-^32^P] ATP was completely removed. The 5′ labelled purified poly(A) free of [γ-^32^P] ATP was used as substrate in a reaction containing 1 µg of rNSs and the 5′ phosphatase activity was assayed as described in the methods section. As shown in Fig. 6A (lanes 2–4), rNSs could cleave the 5′ phosphate from end labelled poly(A) in the absence of ATP and the reaction was inhibited by the addition of 1 mM ATP (lane 5–7). However, control ATPase (Sigma) previously tested to be active for the hydrolysis of ATP, was unable to cleave the 5′ phosphate from the end labelled poly(A) (Fig. 6A lane 8). These results suggest that rNSs indeed possesses a novel 5′ phosphatase activity as well. AMP inhibits the 5′ monophosphatase activity but not ATPase activity. Therefore the rNSs was incubated with increasing concentrations of AMP (1, 2 and 3 mM) and the 5′ phosphatase activity was monitored (Fig. 6B lane 3–5). The 5′ phosphatase activity was completely inhibited by 3 mM AMP (Fig. 6B lane 4). Further, immunodepleted rNSs (Fig. 6B, lane 7) or heat denatured rNSs (Fig. 6B, lane 8) did not cleave the end labelled poly(A) confirming that the cleavage was indeed due to the addition of rNSs. 5′ labelled poly(A) was treated similarly with Calf intestinal alkaline phosphatase (MBI Fermentas) and was used as positive control (Fig. 6B lane 6). siRNA (siblue, purchased from Dharamacon), ssDNA (D159A sense primer), dsDNA (D159A sense and antisense primers annealed) were also 5′ end labelled and used as substrates for 5′ phosphatase activity of rNSs. Interestingly, rNSs could cleave the 5′ phosphate from end labeled dsDNA and ssDNA (Fig. 7A) but not from 5′-end labeled siRNA (Fig. 7C). ATP inhibited the 5′ phosphatase activity even when 5′ end labelled dsDNA or ssDNA were used as substrate (Fig. 7 B). siRNA failed to act as substrate probably because its 5′ end is recessed. To test this possibility, dsRNA prepared by annealing sense and antisense transcript as described in methods section, was used as substrate and indeed rNSs could remove the 5′ phosphate from dsRNA (Fig. 7D). Next, we wanted to examine whether active site residues for ATPase and phosphatase activity are the same or different. Therefore, the D159A and K189A mutants described earlier were purified and their 5′ phosphatase activities were measured with 5′ end labelled poly(A) as substrate along with the wild type enzyme as control. The fractional cleavage by these three proteins (1 µg) at saturating concentration of poly(A) (3 nM) is shown in Fig. 6C. As apparent, D159A mutant showed 20% decrease in activity whereas K189A mutant was as active as the wild type enzyme. These results demonstrate that K189 is not an essential residue for 5′ phosphatase function whereas it is absolutely important for ATPase function.

## Discussion

The results presented in this paper demonstrate for the first time that NSs of GBNV-To (K) is a bifunctional enzyme. Bioinformatic analysis revealed the presence of Walker motif A and B. However, the Walker motif B precedes the motif A in this protein (Fig. 1). It was of interest to examine if rNSs could bind to NTPs and cleave them. As shown in Fig. 3, rNSs exhibited the Poly(A) stimulated ATPase activity. The activity was tenfold higher in the presence of poly(A) compared to that without poly(A). None of the NSs proteins from *bunyaviridae* family have been shown to possess this enzymatic activity. The product was not formed when immunodepleted rNSs was used, which ruled out the possibility of any contaminating bacterial protein (ATPase) co-purifying with rNSs. Further, rNSs could hydrolyze other NTPs and dATP (Fig. 5 and table 1) and the ATPase activity of rNSs was inhibited by an ATP analog {adenosine 5′-(β, γ imido) triphosphate} (Fig. 4C). These results suggested that NTPase/dATPase activity is an intrinsic property of rNSs. On the basis of structural data, it has been suggested that the binding of nucleic acid induces conformational changes in the protein, which, in turn, stabilizes the bound ATP molecules in a conformation that is required for rapid hydrolysis [22], [23]. It is therefore reasonable to suggest that the observed stimulatory effect of poly(A) on GBNV NSs ATPase activity may reflect similar conformational changes. Point mutation in Walker motif B (D159A) reduced the NTPase activity (Fig. 5). NTPase activity is metal ion dependent. The D159, like in other NTP binding proteins, is probably responsible for co-ordination of the metal ion and therefore mutation of this residue affects the NTPase activity. However, the mutation of K189 to A abolished the NTPase activity completely. The conserved lysine residue in the Walker motif A is suggested to be important for interaction with β, γ phosphate of NTP [21]. Therefore, mutation of this residue renders the protein inactive. The lysine residue in the GKT motif is shown to be critical for ATP hydrolysis in many other ATPases as well. For example, the mutation of lysine to glutamine in eIF-4A protein resulted in severely reduced ATP binding, and complete loss of ATPase activity [24]. Gross and Shuman [25] reported that an alanine substitution at the lysine residue of the GKT motif of NPH II decreased its NTPase activity by 1/20 of that of the wild type protein in the presence of RNA or DNA cofactors. The difference in the mutational effects at the same site in eIF-4A and NPHII could be a result of the nature of each protein and sensitivity of the specific experiments. Similar mutation has been performed with NS3 protein of *Dengue virus type* 2 [26] and HCV, which abolished the ATPase activity [27].

In addition to NTPase and dATPase activity, rNSs also exhibited the nucleic acid 5′ phosphatase activity (Fig. 6A) that was inhibited by AMP (Fig. 6B). The rNSs could remove the 5′ Phosphate from ssRNA (Fig. 6A), ssDNA, dsDNA and dsRNA (Fig. 7A, B and D). However, it failed to remove the phosphate from siRNA in which the 5′ end is recessed (Fig. 7C). The 5′ α Phosphatase activity of rNSs was inhibited by ATP. It is possible that ATP acts as the competitor for the binding of the substrate. Interestingly, K189A mutant which was completely inactive as far as the ATPase activity was concerned, was fully active and could remove the 5′ phosphate from 5′ end of the nucleic acid (Fig. 6C). As mentioned earlier, the lysine in the GKT motif is involved in the binding of β, γ phosphate for positioning and cleavage of γ phosphate. However, the rNSs removes the α phosphate from the 5′ end labelled nucleic acid. Therefore, the mutation K189 to A did not result in loss of 5′ phosphatase activity. In the D159A mutant, only a partial decrease (20%) in activity was observed for 5′ phosphatase activity suggesting that this reaction could also be metal ion dependent.

Similar kind of poly(A) stimulated NTPase/dATPase activity has been observed in the case of NS3 protein from *West Nile flavivirus* [*28*], p80 protein from *Pestivirus*, *Bovine Viral Diarrhea virus* [16] gene4 protein from Bacteriophage T7 [29], nucleoside triphosphate phosphohydrolase I (NPH1), NS3 protein from *Hepatitis C virus*, *Yellow fever virus*, *Dengue virus type 2* [16], [17], [18] and Large T antigen from S*imian virus* [16], [30], [31]. Most of these proteins also exhibit the nucleic acid unwinding activity [32], [33], [34], [35], [36]. However, none of the plant viral proteins have been shown to exhibit such activities.

There is only one report on the *in vivo* function of NSs from *Tomato spotted wilt virus*, the type member of Tospovirus genus, as a suppressor of PTGS [3]. There has been no further work on this protein. RNA silencing or PTGS is a major strategy by which plants mount defense responses against molecular parasites such as viruses [37], [38]. The RNA silencing pathway involves the initial processing of viral dsRNA into small interfering RNA (siRNA) duplexes of length 21–25 nt by DICER or its homologs. One of the strands of the siRNA incorporated into the RNA induced silencing complex (RISC) specifically targets viral mRNA with a complementary sequence for degradation [39]. Viruses evade such a defense response by expressing viral suppressors of RNA silencing (VSRs). VSRs encoded by viruses of different families and genera [40], [41] share no homology at the level of primary sequence and the mechanisms by which they inactivate the PTGS are different. The results presented in this manuscript implicate that NSs might act as a suppressor of PTGS by removing 5′ phosphate from dsRNA, the substrate for DICER. It has been observed that DICER does not recognize the dephosphorylated dsRNA[42] and therefore removal of the 5′ phosphate by NSs could eventually block the PTGS pathway. Interestingly, even in some animal viruses dephosphorylated viral RNA fails to induce the Interferon pathway [43], [44] thereby suppressing the host defense mechanism. Further, ATP is required for the PTGS pathway. The ATPase activity of NSs might result in the suppression of this pathway. The ATPase and 5′ phosphatase function of NSs could also be of functional importance in the replication and transcription of the virus.

## Supporting Information

Figure S1 Secondary structure prediction of NSs- The secondary structure of NSs was predicted using Expasy proteomic server. Motif search revealed the presence of Walker A and B motifs (boxed residues).
(4.81 MB TIF)

Figure S2 Optimizing reaction conditions for the ATPase activity of NSs- (A) Time course of ATP hydrolysis; 2 nM of [javascript:app(‘lower case gamma’)32P] ATP was incubated with 1.3 µg of NSs protein for various time intervals (0, 5, 10, 15, 20, 25 and 30 minute) at 25°C. The reaction was stopped at each time point by the addition of 5 mM of EDTA to the reaction mixture. The fractional cleavage of ATP was calculated and plotted as a function of time. Each point represents the average of three experiments. (B) Effect of MgCl2concentration on ATP hydrolysis; ATPase reaction was carried out using 2 nM of [javascript:app(‘lower case gamma’)32P] ATP, 1.3 µg of NSs protein and with increasing concentration of MgCl2 (0.5, 1, 1.5, 2.0, 2.5, 3.0, and 3.5 mM) for 30 minute at 25°C. The fractional cleavage of ATP was calculated and plotted as a function of MgCl2 concentration. Each point represents the average of three experiments. (C) Inhibition of ATPase reaction by EDTA; ATPase reaction was carried out using 2 nM of [javascript:app(‘lower case gamma’)32P] ATP, 1.3 µg of NSs protein and with increasing concentration of EDTA (0, 1, 3, 4 and 5 mM) for 30 minute at 25°C. The fractional cleavage of ATP was calculated and plotted as a function of EDTA concentration. Each point represents the average of three experiments. (D) Effect of pH on ATP hydrolysis; ATPase reaction was carried out using 2 nM of [javascript:app(‘lower case gamma’)32P] ATP, 1.3 µg of NSs protein at various pH values (6.5, 7.0, 7.5 and 8.0) for 30 minutes at 25°C. The fractional cleavage of ATP was calculated and plotted as a function of pH. Each point represents the average of three experiments.
(3.67 MB TIF)
