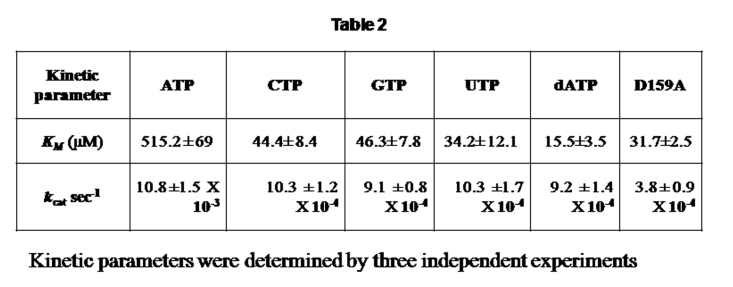# Correction: NSs Encoded by Groundnut Bud Necrosis Virus Is a Bifunctional Enzyme

**DOI:** 10.1371/annotation/82e3c2dd-e7e5-4267-88e5-19d77d5e58e7

**Published:** 2010-04-13

**Authors:** Bhushan Lokesh, Panigrahi R. Rashmi, Bhat S. Amruta, Dharmaiah Srisathiyanarayanan, Mathur R. N. Murthy, Handanahal S. Savithri

A row of data was inadvertently left out. Please view the corrected Table 2 here: 

**Figure:**