# Supplementary figures and images for: NSs Encoded by Groundnut Bud Necrosis Virus Is a Bifunctional Enzyme
Source: PLoS One. 2010 Mar 18;5(3):e9757. doi: 10.1371/journal.pone.0009757 (PMC2841200; doi:10.1371/journal.pone.0009757)

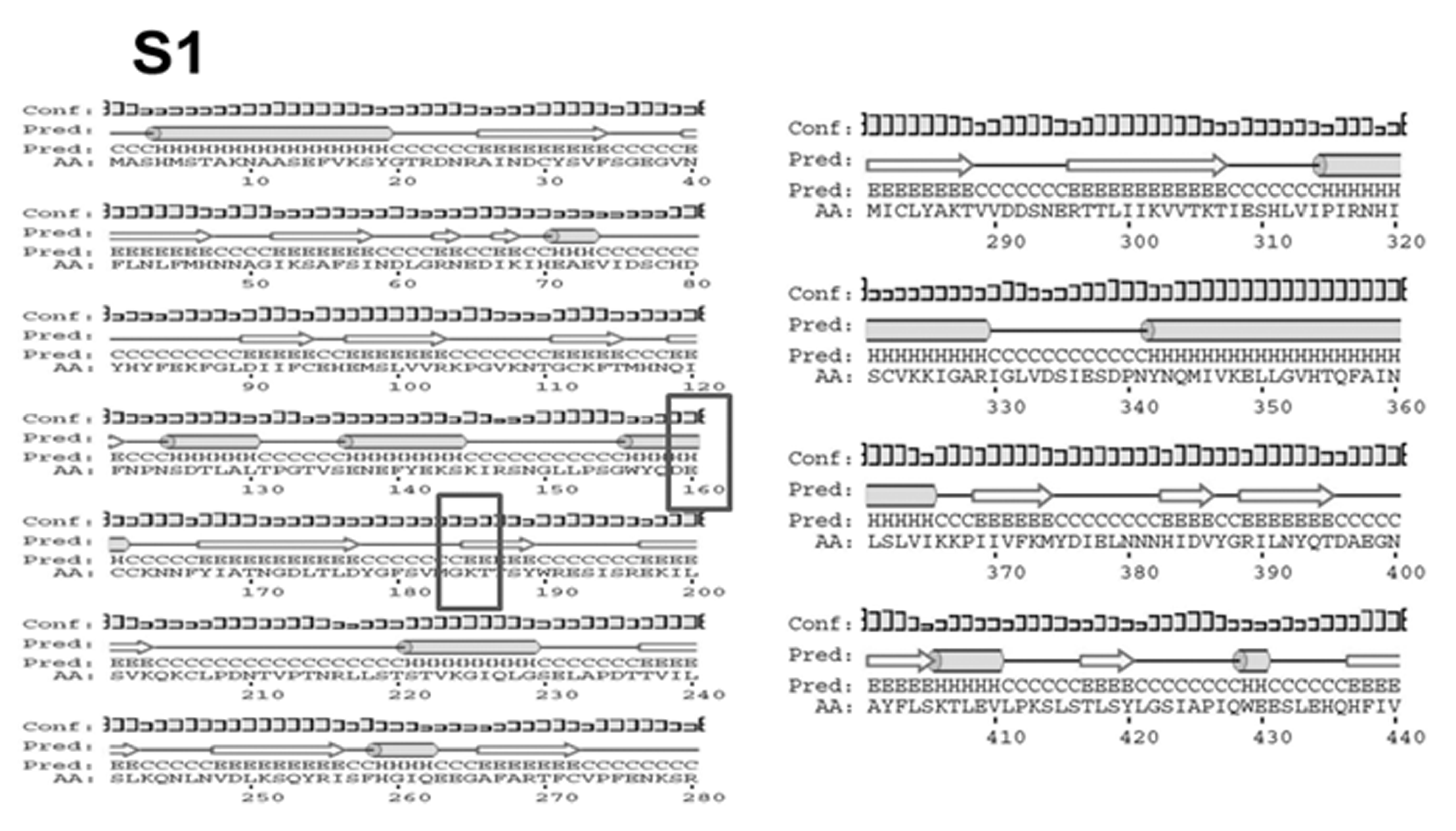

Supplement: Figure S1 — Secondary structure prediction of NSs- The secondary structure of NSs was predicted using Expasy proteomic server. Motif search revealed the presence of Walker A and B motifs (boxed residues). (4.81 MB TIF) [file pone.0009757.s001.tif]

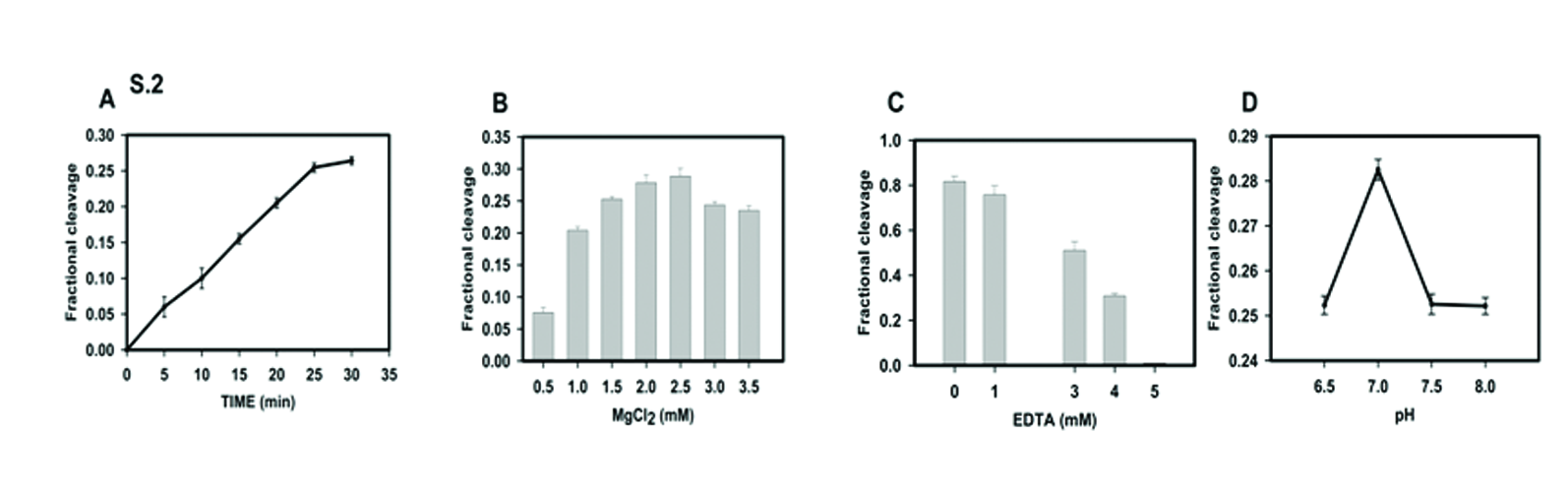

Supplement: Figure S2 — Optimizing reaction conditions for the ATPase activity of NSs- (A) Time course of ATP hydrolysis; 2 nM of [javascript:app(‘lower case gamma’)32P] ATP was incubated with 1.3 µg of NSs protein for various time intervals (0, 5, 10, 15, 20, 25 and 30 minute) at 25°C. The reaction was stopped at each time point by the addition of 5 mM of EDTA to the reaction mixture. The fractional cleavage of ATP was calculated and plotted as a function of time. Each point represents the average of three experiments. (B) Effect of MgCl2concentration on ATP hydrolysis; ATPase reaction was carried out using 2 nM of [javascript:app(‘lower case gamma’)32P] ATP, 1.3 µg of NSs protein and with increasing concentration of MgCl2 (0.5, 1, 1.5, 2.0, 2.5, 3.0, and 3.5 mM) for 30 minute at 25°C. The fractional cleavage of ATP was calculated and plotted as a function of MgCl2 concentration. Each point represents the average of three experiments. (C) Inhibition of ATPase reaction by EDTA; ATPase reaction was carried out using 2 nM of [javascript:app(‘lower case gamma’)32P] ATP, 1.3 µg of NSs protein and with increasing concentration of EDTA (0, 1, 3, 4 and 5 mM) for 30 minute at 25°C. The fractional cleavage of ATP was calculated and plotted as a function of EDTA concentration. Each point represents the average of three experiments. (D) Effect of pH on ATP hydrolysis; ATPase reaction was carried out using 2 nM of [javascript:app(‘lower case gamma’)32P] ATP, 1.3 µg of NSs protein at various pH values (6.5, 7.0, 7.5 and 8.0) for 30 minutes at 25°C. The fractional cleavage of ATP was calculated and plotted as a function of pH. Each point represents the average of three experiments. (3.67 MB TIF) [file pone.0009757.s002.tif]
